# Ocular Ultrasonography: A Useful Instrument in Patients with Trauma Brain Injury in Emergency Service

**DOI:** 10.1155/2019/9215853

**Published:** 2019-01-21

**Authors:** Julie Natalie Jimenez Restrepo, Oscar Javier León, Leonardo Alexander Quevedo Florez

**Affiliations:** ^1^Emergency Department, Third Year Resident of Emergency Medicine, Hospital Universitario San Ignacio, Pontificia Universidad Javeriana, Colombia; ^2^Emergency physician, Emergency Department, Hospital Universitario San Ignacio, Pontificia Universidad Javeriana, Colombia; ^3^Emergency Medicine Physician, Pontificia Universidad Javeriana, Fellowship in Critical Care Medicine, Universidad de la Sabana, Bogotá, Colombia

## Abstract

The measurement of the optic nerve sheath by ocular ultrasonography might be an indirect method to assess the quickly increase of the intracranial pressure in patients with moderate trauma brain injury, taking into account that an important proportion of these could develop the increase of the intracranial pressure in a hospital-acquired way. Therefore noninvasive, reliable, and convenient techniques are needed making the ocular ultrasonography a useful tool, due to the invasive monitoring elements' problems and the poor access to measure the intracranial pressure in emergency services. In spite of the limitations and few studies that exist to consider it as a possible early detection, this technique could work as a noninvasive one in the case that could not be possible to do invasive monitoring or when it is not recommended.

## 1. Introduction

The elevation of the intracranial pressure (ICP) is a diagnostic challenge in the emergency service and a potentially lethal complication in patients with trauma brain injury when entering to the emergency service. These patients need an immediate attention to avoid future complications [1].

Physical exam which includes the patient's neurologic assessment is the first diagnosis tool, and unfortunately delayed changes might be shown after the physical exam and, thus, it is determined to use other tools for the ICP increase prompt diagnosis [1].

First, computed tomography and nuclear magnetic resonance are used as initial methods of idiopathic intracranial hypertension diagnosis (IIH) and its complication; however, they are not the appropriate methods to the critical patient monitoring since they are not always available in emergency situations and they are too expensive to lead to [2].

The optic nerve sheath ultrasonography measure seems to be a valid alternative method with several advantages like the accessibility, opportunity, cheapness, monitoring (since it is profitable and can be repeated) and not being invasive in critical patient context since morbidity and mortality can increase especially in the emergency services and intensive care unit (ICU) [3, 4].

Helmke and Hansen were the first to find out the ultrasonography to detect IIH in corpses in 1996. Since then, ocular ultrasonography has been used in bedside ocular ultrasound patients (BOUS) for the early detection of IIH and numerous studies have tried to show its use and advantages in emergency services [5].

**Figure 1:**
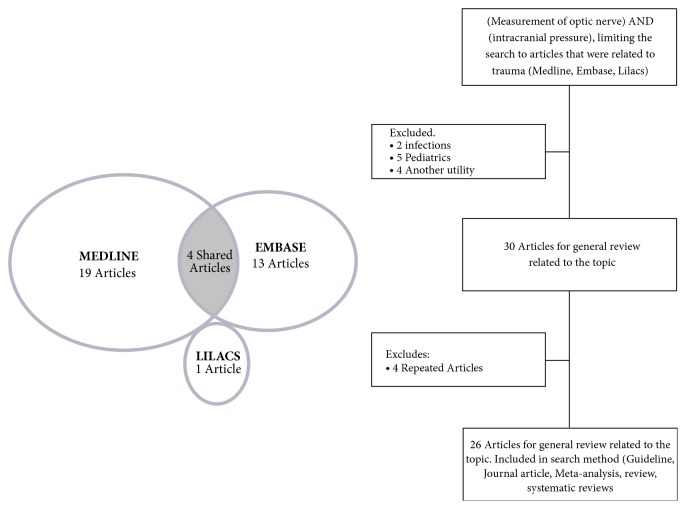
Search methods in databases.

A search was made in the main databases (Medline, Embase, and Lilacs), focusing the search methods on (Measurement of optic nerve) AND (intracranial pressure), limiting the search to articles that were related to trauma. The commands for Lilacs were as follows: (tw: (measurement of optic nerve)) AND (tw: (intracranial pressure)) AND (tw: (trauma)) OR (tw: (injury)), obtaining a single article, the search command of Medline was ((“Measurement (Lond)” [Journal] OR “Measurement (Mahwah NJ)” [Journal] OR “measurement” [All Fields]) AND (“optic nerve” [MeSH Terms] OR (“optic” [ All Fields] AND “nerve” [All Fields]) OR “optic nerve” [All Fields])) AND (“intracranial pressure” [MeSH Terms] OR (“intracranial” [All Fields] AND “pressure” [All Fields] ) OR “intracranial pressure” [All Fields]) AND (“wounds and injuries” [MeSH Terms] OR (“wounds” [All Fields] AND “injuries” [All Fields]) OR “wounds and injuries” [All Fields] OR “injury” [All Fields]) AND (“humans” [MeSH Terms]) obtaining 23 articles and the search command of Embase was (“measurement of optic nerve”: ti, ab, kw AND “intracranial pressure”: ti, ab, kw AND “injury”: ti, ab, kw), obtaining 17 articles, achieving a total of 41 articles. 5 articles were excluded because they were children, 2 because they were infectious processes rather than trauma or general population, 4 another utility, in addition to 4 articles repeated in the different bibliographical bases for a total of 26 articles for general review related to the topic (Figure 1).

## 2. Optic Nerve Anatomy

The optic nerve is the cranial nerve II composed by fiber tract with 1,2 millions of axons surrounded by unsheathed meninges that transmit all visual information to the central nervous system. It is composed of retinal ganglion cells and glial cells. It is detached 3 mm towards the middle and 1 mm lower from the eyeball to the posterior pole. Then it is conducted postero-medial through the cranial cavity and ends in the anterolateral angle, which belongs to the optic chiasm and measuring 5 cms approximately [6].

The optic nerve is surrounded by a cerebrospinal fluid and it is enclosed in a sheath that is an dural sheath extension. That intraorbital portion can be visualized with ultrasound. This uninterrupted connection provides a direct ICP transmission to the optic nerve sheath since there is communication among the perineural space, the cerebrospinal fluid and the subarachnoid space, hence, when CSF pressure increases, it moves the intraorbital sheath and it gets longer. According to this it is possible to establish that the measurement in the optic nerve sheath diameter changes provide information about the ICP changes [7].

## 3. Intracranial Pressure

It is known as the pressure increase in intracerebral levels as the result of a neurologic injury which can cause high mortality and morbidity in patients with trauma brain injury [8].

When a pressure increase is shown, several methods have been used for measuring ICP such as the radiologic imaging or invasive techniques, but the “gold standard” for the diagnosis of elevated ICP is “pressure measure in a invasively way by intracranial catheters connecting to a pressure transducer” [9]. It is considered as a highly invasive procedure and may result in severe complications such as infection, hemorrhage, and malfunction [10, 11].

A prospective study done in the Intensive Care Unit (ICU) with intracranial pressure monitoring showed a strong correlation between the optic nerve sheath diameter (over 5 mm) and the ICP (greater than 20 cms H20) [12]. Thus, it was considered that the optic nerve sheath measuring through ocular ultrasonography is a useful method, since it is noninvasive and dynamic in patients with IIH, which allows reducing the inherent complications related to invasive methods [7, 13, 14].

## 4. Ocular Ultrasonography: Technique

**Figure 2:**
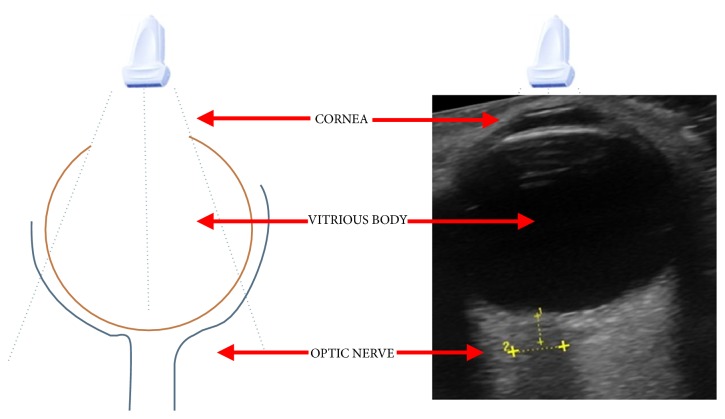
Representation of the anatomy under ultrasound vision.

To do an ultrasonography ocular in emergency service the patient must to be in supine position with his eyes closed (eyes can be covered with an insulating element to avoid the contact between the eye and the gel which is the best signal conductive to get a better image). This procedure is done with a linear transducer which is placed on the eyelid and is pressed smoothly on the eyeball. It is done in 3 planes: axial horizontal, axial vertical, and vertical transverse [5, 15] (Figure 2).

**Figure 3:**
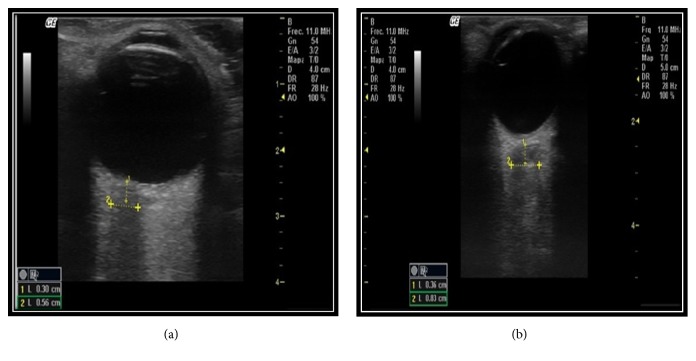
Ocular ultrasonography, mode B, with measurement of the optic nerve sheath, suggestive of endocranial hypertension, (a) transverse view, and (b) sagittal view.

A slipping is done to check every important part of the eyeball. In the ultrasound machine B mode, we are able to check the sheath of the eye that in normal ranges must be around 3 mm right behind the retina since it is the highest point where we can check in an enhanced way the second disorders in the ICP increase [16] (Figures 3(a) and 3(b)).

## 5. Ocular Ultrasonography and Intracranial Pressure

In the last decades the ultrasonography has proved to be a useful tool for many pathologies that require a proper diagnosis and handling in the emergency service [13].

The American College of Emergency Physicians (ACEP) has considered the use of bedside emergency ultrasound as a vital aspect in the visual exam of the patients, being applicable in patients with suspected retinal detachment initially [12].

Other ocular ultrasonography use in emergency services that has been demonstrated as a promising tool is the measure of the optic nerve in patients with traumatic brain injury in patients with suspected Idiopathic intracranial hypertension (IIH) (Figure 3(a)), since the invasive neurological monitoring is improbable to be done in emergency services unlike ICU. That is why the ocular ultrasonography is considered as an efficient tool for the emergency room doctors. Besides, it is considered as a noninvasive, dynamic, and repetitive procedure that provides timely information in the monitoring of these patients [2, 13, 17].

A prospective study showed the ocular ultrasonography benefit in neurosurgical patients, it proved a positive correlation between the diameter of the optic nerve sheath and the ICP measurements. 95% sensitivity and 80% specificity were the most specific in those patients with a history of traumatic brain injury [18].

Other study was researched with 31 patients with posttraumatic brain injury and a Glasgow coma scale lower than 8 that required sedation and ICP monitoring; patients with ICP higher than 20 mmHg were compared to others with normal ICP. It showed that the ocular ultrasonography is a useful tool in the early detection in ICP increase because values higher than 5 mm of the optic nerve sheath were correlated with ICP higher than 20 mmHg with sensitivity and 100 negative predictive values [19, 20].

A study done in 2001 by Cammarata et al., with ICU patients with severe traumatic brain injury considered as Glasgow coma scale lower than 8, established that the diameter of optic nerve sheath was close to 7mm when those patients had CIP higher than 20 mmHg. When CIP was lower than 20 mmHg, the diameter continued with 5.5 mm being an approximate value established in control group (patients in the same ICU without traumatic brain injury and no suspected of IIH) [21, 22].

In 2007, Tayal et al. did an observational study that showed a sensitivity of 100% in the measure of the bilateral optic nerve sheath in contrast of the simple skull tomography, a specificity of 63%, a positive predictive value of 30%, and a negative predictive value of 100%. This study showed multiple limitations due to the type of study, sample size determination and the suitability of the sampling method. But it did allow showing an initial approach of possible advantages in this emergency service intervention. Moreover, it established the learning curve to do an ocular ultrasonography that is fast and does not mean limitations to introduce this technique in clinical practice [1].

Being ultrasonography an operator, in 2015, Ozturk et al. did a study in which 4 specialists in emergency service were chosen to do ocular ultrasonography in healthy patients and to determine the interobserver correlation. Two axial and longitudinal measurements were done and 960 measurements were contrasted. The results of this prospective study did not show any difference between measurements, but they did among specialists [9, 23].

## 6. Special Conditions

Dip et al. did a study to assess the optic nerve sheath diameter changes in obese patients. The results showed that it is statistically significant that the optic nerve sheath diameter differs among obese and nonobese patients, which means possible false positives in increased IIH in obese patients with 5 mm optic nerve sheath diameter as has been established in medical literature. It should be noted that writers did not clearly ascertain on the basis of which parameters established the difference between obese and nonobese, and it was indirectly portrayed as a reference point the limit of the body mass number higher than 30 Kg/m^2^ [24].

Contrasting the optic nerve sheath diameter of healthy pregnant women with patients with diagnosis of pre-eclampsia, the diameters, respectively, vary from 4.5 mm to 5.4 mm with a 96% confidence range value and a p value statistically significant. In addition, after the birth, 19% of the patients with preeclampsia had the optic nerve sheath diameter over 5.8 mm [25].

In 2015, a study case in children was checked and it confirmed that ocular ultrasonography was an examination that might provide 2 signs related to papilledema; there are Optic Disc Elevation (elevation of one or more mm above the retina of entry of the optic nerve) and the Crescent Sign Using Point-of-Care as the fluid that surrounds the optic nerve when it is seen in transverse windows with vertical orientation that demonstrated its relationship with an increase in ICP [18, 26].

## 7. Discussion

The patient's assessment with trauma brain injury with ICP increase is a challenge in the emergency service. Many of these patients display serious complications and an early diagnosis might settle therapeutic measures that could contribute to a better survival. The physical examination is not enough in the emergency services to assess the ICP increase in patients with trauma brain injury. Furthermore, these patients can show states of consciousness, being under sedation or with respiratory support that makes a limit among the interrogation and the neurological examination.

There are many alternative diagnoses to assess the intracranial pressure elevation as the ophthalmological exam looking at the papilledema and the changes in pupils; nevertheless, these ones have a belated submission. Likewise, the diagnostic imaging like the computed axial tomography and the nuclear magnetic resonance is not available in most of the emergency services, and it also requires time and sometimes can cause higher morbidity.

The Bedside Ultrasonography is an excellent alternative to assess patients with intracranial pressure elevated in the emergency services, taking into account that is a noninvasive and fast procedure that does not require preparation and patient's transportation. The proof allows confirming that the ultrasonography is a useful tool to establish the ICP elevation by means of measure of the optic nerve sheath through ocular ultrasonography. Currently there is no evidence enough to prove that ocular ultrasonography is a selection tool in patients with IIH since many studies are observational and those that were made with different samples did not allow extrapolating the results because they were too small.

Another situation that must be taken into account is the type of patient and why the medical condition IIH is suspected. Inside the evidence, most of the participants showed trauma brain injury and, also, this made it possible to validate the use of this tool in other conditions for which we have no information. In addition, it is important to take into account that special situations as obese patients, pregnancy, and pediatrics population might affect measurements.

When comparing ICP measurements in an invasive way with the optic nerve sheath measure through ultrasonography, the results show a good correlation among the values and the ICP estimated. Same results were achieved with the computed axial tomography measuring the negative predictive value and sensibility, but these studies show many methodological limitations that do not allow coming up with a recommendation about it.

Finally, regarding the follow-up patients and the comparison with many measurements among different specialists, it is clear that the ultrasonography continues being a dependent operator method and a diagnosis tool that can have a unique value in the patient's attention; it also must have a clinical correlation to be able to determine behaviors with the results obtained.

## 8. Conclusion

Despite the low evidence ocular ultrasonography can be used as a diagnostic tool in IIH for those patients with trauma brain injury in emergency service and intensive care. There exists a lack of studies with the ability to establish the daily use of this tool and target population in institutions.

## Data Availability

Data sharing is not applicable to this article as no datasets were generated or analyzed during the current study.
